# Interleukin-6 and interleukin-10 gene polymorphisms and recurrent pregnancy loss in Romanian population

**Published:** 2014-09

**Authors:** Camil L. Bohiltea, Viorica E. Radoi

**Affiliations:** *Medical Genetics Department, UMF Carol Davila, Bucharest, Romania.*

**Keywords:** *Interleukin*, *Pregnancy loss*, *Polymorphisms*

## Abstract

**Background:** Approximately 10-14% of the clinically acknowledged pregnancies end with spontaneous abortion at Caucasian population. Possible immunologic causes of recurrent miscarriages have been extensively researched. The change in the cytokines balance synthesis in favor of those synthesized by Th2 cells with an increase of interleukin 6 (IL6) and interleukin10 (IL10) secretion is considered essential for maintaining a normal pregnancy.

**Objective:** The study objective was to establish an association between interleukin 6 and 10 genes polymorphisms and etiology of recurrent pregnancy loss.

**Materials and Methods: **The genetic polymorphism of interleukin 6 and 10 genes were studied by PCR-RFLP in the DNA of 69 women with recurrent pregnancy loss and 64 control women with at least one successful pregnancy and without known pregnancy loss. Statistical analysis was performed using Fisher test and differences were considered statistically significant with a p<0.05.

**Results: **Our results demonstrated a role for -819 C/T but not for -592 C/A IL10, -1082 A/G IL10 and -174G/C IL6 polymorphisms in idiopathic recurrent spontaneous abortion (RSA) in Romanian population. Frequency of genotype -592 CC/-819 CC was higher in the control group than in experimental group (p=0.005). In contrast, genotype -592 AC/-819 CT was more frequent in the experimental group (p=0.05). In this study we have not detected genotype -174 C/C in IL6 gene in patients with spontaneous abortions, nor in the control group.

**Conclusion:** This study demonstrated a possible association between IL-10 -819 C/T promoter polymorphism and idiopathic RSA among Romanian patients.

## Introduction

Approximately 10-14% of the clinically acknowledged pregnancies end with spontaneous abortion at Caucasian population. In many of these cases the etiology remains unclear (1). Possible immunologic causes of recurrent miscarriages have been extensively researched (2). Previous studies have shown that the most important mechanism of immune mediated pregnancy failure is immune activation at the maternal- fetal interface (3). Stimulation of decidual immune cells with increased pro-inflammatory cytokine production cause direct damage the embryo and the placenta (4). 

Successful pregnancy has been demonstrated to be T helper 2 type phenomenons with a decreased T helper 1 activity (4). Placental and decidual tissues from normal pregnancies express an array of pro and anti-inflammatory cytokines (5). An abnormal Th1 type cellular immune response is a recent hypotesis for immunological reproductive failure in women (6). In cases of pregnancy loss, deregulation of the mother’s immune system could be responsible for the failure where the implanting embryo is recognized as foreign and is thus rejected (6, 7). Natural killer cells floading the uterus at the time of implantation carries receptors that interact with HLA-C, HLA-E on surface of trophoblast, triggering the production of cytokines that help trophoblast to invade or limit the extent to which it invades (5). 

The first studies on spontaneous recurrent miscarriage associated abnormal immune reactivity in the context of Th1-Th2 paradigm demonstrate in vitro that trophoblast antigens activate lymphocytes to produce embryotoxic cytokines i.e. TNF-α, IFN-γ and IL-2 (4, 5). TNF-α and IFN-γ inhibit embryonic and fetal development as well as the proliferation of human trophoblast lines as both these cytokines are cytotoxic to embryonic fibroblast like cells. It is also demonstrated that IL-2, TNF-α and IFN-γ together terminate normal pregnancy when injected (4, 5). The change in the cytokines balance synthesis in favor of those synthesized by Th2 cells with an increase of interleukin 6 (IL6) and interleukin10 (IL10) secretion is considered essential for maintaining a normal pregnancy (7).

Several studies have analyzed the possible correlations between 3 polymorphisms present at the level of the promoter of the codifying gene for IL10 and the abortion disease, namely polymorphisms -592C/A, -819C/T and -1082A/G. Conclusions varied based on the researched population groups (8-10). Relationship between the polymorphism -174G/C in the gene that codifies for IL6 and several conditions (anomalies of the lipid metabolism, autoimmune disorders) has already been proven (11, 12). Spontaneous recurrent miscarriages represent an important medical problem. Numerous researches have been performed for discovering the possible causes of abortion disease; however, at a significant percentage of patients, the exact cause of spontaneous abortions could not be identified. Immunological factors play an important role in carrying and the physiological evolution of a pregnancy. Few studies have been conducted to present to establish relationships between the codifying genes for cytokines and the abortion disease. 

This study aims at analyzing polymorphisms in the codifying genes for IL6 and IL10 (polymorphisms controlling the quantity of synthesized cytokines) and the influence of these polymorphisms over the pregnancy evolution at the Romanian population; this is the first study to be conducted in our country (13).

## Materials and methods

This was a case-control retrospective study and has been performed in Bucharest at Department of Maternal and Fetal Medicine Life Memorial Hospital between 2008-2011. Cases comprised 69 women with two or more recurrent spontaneous abortions of unknown etiology. The control lot comprised 64 volunteer women, with no history of spontaneous abortions and at least one pregnancy carried to term without any complications.

Exclusion criteria included anatomical abnormalities, previously known systemic disease, endocrine disorders, previous venous or arterial thrombosis or a family history of thromboembolism. Chromosomal abnormalities were ruled out (karyotype) before inclusion in the study. As infection was linked with recurrent spontaneous abortion (RSA), all subjects included were confirmed to be negative for the TORCH agents *Toxoplasma gondii*, rubella, cytomegalovirus (CMV), herpes simplex viruses (HSV-1 and HSV-2), varicella zoster virus (VZV) and human immunodeficiency virus (HIV-1 and HIV-2). Transvaginal ultrasound was performed to confirm spontaneous abortion. The study was performed based on an informed consent and was approved by UMF Carol Davila Bucharest Ethics Committee.

The demographic characteristics of the patients were presented in table I. DNA was extracted from peripheral blood using standard techniques (PROMEGA kit). The sequence amplification was performed by way of PCR technique (polymerase chain reaction). For interleukin 6 and 10 genes polymorphisms PCR conditions comprised an initial denaturing step at 95º C for 15 min, followed by 35 cycles of 95º for 1 min, 56^o^C for 2 min and 72^o^C 1 min and a final extension at 72^o^C for 10 min.

**IL-10 gene polymorphisms**

**Figure 1:**
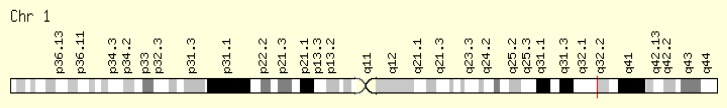
Interleukin 10 gene on chromosome 1, 1q32.2 (qhr.nlm.nih.gov.)

Gene for interleukin 10 is located on chromosome 1 (Figure 1). Genomic DNA was extracted from peripheral blood leukocytes by the Promega Extraction Kit. Cytokine gene polymorphisms were determined by PCR- RFLP. For the -592C/A SNP (rs 1800872), DNA was amplified using a common forward primer (5′ GCTCACTATAAAAATAGAGACGG -3′) and specific reverse primers for the C (5′-CTGGCTTCCTACAGG- 3′) and the A (5′-GAC TGGCTTCCTACAGT-3′) alleles. 

Similarly, for the -819C/T SNP (rs1800871), DNA was amplified using a common forward primer F:5’GACAACACTAC TAAGGCTCCTTTGGG A3’ and reverse primer R:5’GTGAGCAAACTGAGGCACAGA AAT3’ and for -1082G/A SNP (rs1800896): forward primer F: 5’GACAACACTACTAAGG CTCCTTTGGGA3’ and reverse primer R:5’GT GAGCAAACTGAGGCACAGAAAT 3’. Positive controls were selected by amplifying and sequencing two regions of the IL-10 promoter. A reagent control without DNA served as negative control.

**IL6 polymorphism**

Gene for interleukin 6 is located on chromosome 7 (Figure II). For the -174G/C SNP (rs 1800795), DNA was amplified using a common forward primer F: 5’GGAGTCACACA CTCCACCT3’ and reverse primer R:5’CTGAT TGGAAACCTTATTAG3’. For positive control we used a known individual heterozygous for C allele. A reagent control without DNA served as negative control. The restriction enzymes and sequences of the restriction sites for IL6 and IL10 gene polymorphisms are presented in table II.

**Statistical analysis**

Statistical analysis was performed to determine odd ratio (OR) and 95% confidence intervals (95%CI) associated with recurrent pregnancy loss, using SPSS software (Statistical Package for the Social Sciences, version 20.0, SPSS Inc, Chicago, Illinois, USA). The p<0.05 was considered to be statistically significant.

## Results

The demographics of the trial persons were indicated in table I. The frequencies of the genotypes/alleles 819 C/T, 592 C/A and 1082 A/G in IL10 gene and 174 C/G in IL6 gene in study and control lot, are presented in tables III, IV. The distribution of IL-10-592C/A, -819C/T genotypes was in Hardy-Weinberg equilibrium among controls. The frequency of IL-10-592A (0.32 vs. 0.25; p=0.06) and C (0.67 vs. 0.75; p=0.50) alleles were similar between patients and controls (Table III). In contrast, the frequency of the IL-10-819 (mutant) T allele (0.32 vs. 0.18; p=0.02; OR=2.09), was higher among patients (Table III). 

With the exception of the -819C/C genotype, which was lower among patients than controls (p=0.003; OR=0.33; 95% CI=0.16-0.67), and 819C/T genotype which was higher among patients than controls p=0.004; OR=2.95; 95% CI=1.43-6.08) the frequencies of IL-10-592C/A genotypes and 819 TT genotype were comparable between patients and controls (Table III). The presence of the genotype -819 CC (homozygous for normal allele) was lower in the experimental lot compared to the control lot and the presence of the genotype -819 CT (heterozygous), but not of genotype -819 TT was higher in the experimental lot compared to the control lot. 

The frequency of the genotypes and allelic frequencies are presented in table IV. No significant differences were registered between the study and the control lot. Both in the experimental lot as well as in the control lot, the CC genotype could not be identified, probably due to detrimental effects over fetal development that are incompatible with survival. The obtained data have shown that there is no association between the presence of allele C in position -174 from the region of the IL6 gene promoter and recurrent pregnancy loss. The next step was to analyze the correlation between different combination of studied polymorphisms and increased risk for recurrent miscarriages.

Frequency of genotype -592 CC (homozygous for normal allele) /-819 CC (homozygous for normal allele) was higher in control group than experimental group (p=0.005). In contrast, genotype -592 AC (heterozygous) /-819 CT (heterozygous) was more frequent in the experimental group (p=0.05). The frequency of the genotype -819 CC (homozygous of normal allele) /-174 GG (homozygous of normal allele) was higher in the control lot compared to the experimental lot. By contrast, genotype -819 CT (heterozygous)/-174 GG (homozygous of normal allele) was more frequent in the experimental lot compared to the control lot. Genotype -1082AG (heterozygous)/-174 GG (homozygous of normal allele) was more frequent in the experimental lot compared to the control lot. 

Otherwise no statistically significant differences between two lots were registered. For polymorphisms -592 C/A şi -819 C/T allelic frequencies are in concordance with the frequencies reported previously at Caucasian population. For polymorphism -1082 A/G allelic frequencies are discordant with those reported at Caucasian population (14). For polymorphism in position -174 G/C in IL6 gene, the allelic frequencies are discordant with the frequencies reported previously at the Caucasian population (15).

**Table I:** Demographic characteristics for patients and control group (BMI report based on weight and height of each person (weight^2^/ height).

	**Cases (mean±SD)**	**Control (mean±SD)**	**p-value**
Age (year)	29.6 ± 6.3	27.9 ± 4.2	0.21
Abortions (number)	3.8 ± 0.9	0.0 ± 0.0	0.01
BMI	25.2 ± 3.6	24.4 ± 2.9	0.001

Statistically significant p-value <0.05.

**Table II:** Restriction sites and enzymes used for IL6 and IL10 gene polymorphisms

**Polymorphism **	**Restriction enzyme**	**Restriction site**	**Restriction fragments**
-174 G/C IL6 (rs 1800795)	Nla III	CATG↓	331 bp, 29 bp
-592 C/A IL10 (rs 1800872)	SspI	AAT ↓ATT	122 bp, 45 bp
-819 C/T IL10 (rs 1800871)	SspI	AAT ↓ATT	219 bp; 24 bp
-1082 G/A IL10 (rs 1800896)	BSeLI	CCNNNNN↓NNGG	290 pb; 25 pb

**Table III:** Genotype and allele frequencies of IL10 gene polymorphisms

					**Genotypes**				**Alleles (frequency)(mean±SD)**	
**IL10-592C/A**					**CC**	**CA**	**AA**		**C**	**A**
	Patients				30 (69)	33 (69)	6 (69)		0.673 ± 0.02	0.326 ± 0.02
	Control				35 (64)	26 (64)	3 (64)		0.750 ± 0.02	0.250 ± 0.02
	P				0.22	0.48	0.49		0.26	0.28
	OR (CI)				0.637 (0.321-1.264)	1.33 (0.674-2.66)	1.93 (0.463-8.0916)		0.688	1.477
**IL10-819C/T**					**CC**	**CT**	**TT**		**C**	**T**
		Patients			28 (69)	37(69)	4 (69)		0.673±0.02	0.326 ± 0.02
		Control			43 (64)	18(64)	3 (64)		0.812±0.02	0.187 ± 0.02
		P			0.003	0.0046	1.00		0.02	0.03
		OR (CI)			0.33 (0.16-0.67)	2.95 (1.435-6.080)	1.253 (0.269-5.820)		1.25	2.09
**IL10-1082A/G**					**AA**	**AG**	**GG**		**A**	**G**
			Patients		7(53)	28 (53)	18 (53)		0.40 ± 0.02	0.60 ± 0.02
			Control		11(64)	24(64)	29 (64)		0.36 ± 0.02	0.64 ± 0.02
			P		0.61	0.13	0.257		0.65	0.66
			OR (CI)		0.73 (0.26-2.04)	1.86 (0.89-3.91)	0.62 (0.29-1.31)		1.18	0.84

Statistically significant p<0.05; OR - odds ratio;CI - confidence interval

**Table IV:** Genotype and allele frequencies of IL16 gene polymorphism

**IL16-174G/C**	**Genotypes**				**Alleles (frequency)** **(mean±SD)**	
	**GG**	**CG**	**CC**		**G**	**C**
Patients	60 (69)	9 (69)	0		0.93 ± 0.002	0.065 ± 0.002
Control	56 (64)	8(64)	0		0.93 ± 0.002	0.062 ± 0.002
P	1,00	1.00			0.90	0.90
OR (CI)	0.9 (0.34- 2.64)	1.05 (0.37- 2.91)			0.95	1.051

Statistically significant p-value <0.05; OR- odds ratio; CI - confidence interval

**Figure 2:**
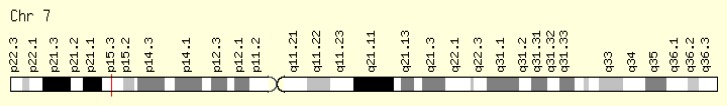
Interleukin 6 gene on chromosome 7, 7p15.3. (qhr.nlm.nih.gov.)

## Discussion

A successful pregnancy depends on maintaining equilibrium between immunity mediated by Th1 cells and that mediated by Th2 cells (16). A predominance of Th2 immunity was observed, that means the stimulation of cytokines synthesis, produced by Th2 cells, among which IL6 and IL10 have an extremely important role. Also, the physiological evolution of pregnancy depends on the reduction of cytokine synthesis produced by Th1 cells as well (17, 18). The cytokine production is under genetic control and in the current study we registered the correlation between the polymorphisms (SNP) present at the level of the codifying for IL6 and IL10 and the abortion disease.

The etiology of recurrent abortions is heterogeneous (infectious, genetic, anatomic, hormonal factors are involved), accentuated by the acquired or birth risk factors. In the current study we established a possible correlation between polymorphism -819 C/T in gene IL10 and recurrent abortions. A study conducted in 2006 has shown the correlation between polymorphisms -592 C/A and -819 C/T and early spontaneous abortions; another study performed on the population in Iran, has demonstrated the existence of the correlation only between the polymorphism -592 C/A and recurrent abortions (9, 14). There were also studies that concluded that there are no correlations between any of the 3 described polymorphisms and the etiology of spontaneous miscarriages. 

Insofar as its production varies as per the specific polymorphism, the role of IL-10 in RSA pathogenesis remains controversial (19, 20). It was suggested that increased IL-10 expression was associated with successful pregnancy, whereas low levels were linked with recurrent fetal loss (21-23). Others suggested the opposite, that enhanced IL-10 production was seen in recurrent pregnancy loss cases compared with fertile women (7, 24, 25).

Others claimed that IL-10, absent in the serum of healthy pregnant women, was detected during abortion and labor (24, 25). These discrepancies may be explained by ethnic differences of the study groups as well as by number variations of the subjects that are included in the research. Although explanation for these discrepancies remains to be seen, the complexity of cytokine balance within the endometrium and decidua, coupled with the influence of maternal hormones, dictates the Th1 and Th2 cytokine balance during pregnancy (26). 

During pregnancy, there is a detectable level of IL6 in maternal serum, that increases at birth (2021), Von Wollf and colleagues found an abnormal expression of IL6 mRNA in endometrium during the mid- secretory phase in women with RPL (22). Others reported significantly higher serum concentration of the Th2 cytokines IL6 and IL10 at normal delivery than in women with RPL. Furthermore, higher IL6 levels were found in women with RPL who had a successful pregnancy as compared with women with RPL who had another abortion (20).

The frequency of allele C was 0.06 in the studied lot compared to 0.40-0.45 at the Caucasian population. Allele C has not been identified in position -174 in a study performed on 388 Japanese men and at only one person in a study performed on 259 Chinese men (23, 24).

## Conclusion

Taking into account the results obtained in this study, as well as the results of previous studies we may conclude that there is not only one genetic factor, but possibly several that are involved in the abortion disease etiology, as favoring factors (24, 25). If the relationship between genetic factors and the immune system disorders is cleared, genetic polymorphisms as the one that is studied may represent markers for selecting the therapeutic options and for counseling patients with recurrent spontaneous abortions. The recurrent spontaneous abortions etiology is a multi-factorial condition with both immune as well we non-immune causes. This study has proven a possible association of polymorphism -819 C/T and the increased frequency of recurrent abortions and the lack of association between polymorphisms IL10-592C/A, -1082A/G, IL6-174G/C and the abortion disease in the studied group.

## Footnote

***This article extracted from Ph.D. Thesis. (Radoi VE)***

## References

[1] Stirrat GM (1990). Recurrent miscarriage; definition and epidemiology. Lancet.

[2] Clark DA, Chaouat G, Wong K, Gorczynski RM, Kinsky R (2010). Tolerance mechanisms in pregnancy: a reappraisal of the role of Class I paternal MHC antigens. Am J Reprod Immunol.

[3] Bidwell J, Keen L, Gallagher G, Kimberley R, Huizinga T, McDermott MF (2001). Cytokine gene polymorphism in human disease: online databases. Genes Immunol.

[4] Bobe P, Kannellopoulus-Langevin C, Bleux C, Voison GA (1986). Modulation of mouse anti-SRBC antibody response by placental extracts II Antigen specificity and regulatory role of B and T cell populations affected by two distinct placental fractions.. J Immunol.

[5] Saini V, Arora S, Yadav A, Bhattacharjee J (2011). Cytokines in recurrent pregnancy loss. Clin Chim Acta.

[6] Bansal AS (2010). Joining the immunological dots in recurrent miscarriage. Am J Reprod immunol.

[7] Raghupathy R (2001). Pregnancy: success and failure within the Th1/Th2/Th3 paradigm. Semin Immunol.

[8] Daher S, Shulzhenko N, Morgun A, Mattar R, Rampim GF, Camano L (2003). Associations between cytokine gene polymorphisms and recurrent pregnancy loss. J Reprod Immunol.

[9] Kamali-Sarvestani E, Zolghadri J, Gharesi-Fard B, Sarvari J (2005). Cytokine gene polymorphisms and susceptibility to recurrent pregnancy loss in Iranian women. J Reprod Immunol.

[10] Babbage SJ, Arkwright PD, Vince GS, Perrey C, Pravica V, Quenby S (2001). Cytokine promoter gene polymorphisms and idiopathic recurrent pregnancy loss. J Reprod Immunol.

[11] Fernandez-Real JM, Broch M, Vendrell J, Richart C, Ricart W (2000). Interleukin-6 gene polymorphism and lipid abnormalities in healthy subjects. J Clin Endocrinol Metab.

[12] Fishman D, Faulds G, Jeffery R, Mohamed-Ali V, Yudkin JS, Humphries S (1998). The effect of novel polymorphisms in the interleukin-6 (IL-6) gene on IL-6 transcription and plasma IL-6 levels, and an association with systemic-onset juvenile chronic arthritis. J Clin Invest.

[13] Carol Davila (2012). Radoi VE Biological markers of poor prognosis in pregnancy outcome- clinical and molecular correlations. Presented for PhD.

[14] Zammiti W, Mtiraoui N, Khairi H, Gris JC, Almawi WY, Mahjoub T (2008). Associations between tumor necrosis factor-alpha and lymphotoxin-alpha polymorphisms and idiopathic recurrent miscarriage. Reproduction.

[15] Olomolaiye O, Wood NAP, Bidwell JL (1998). A novel NlaIII polymorphism in the human IL-6 promoter. Eur J Immunogenet.

[16] Chaouat G, Zourbas S, Ostojic S, Lappree- Delage G, Dubanchet S, Ledee N (2002). A brief review of recent data on some cytokine expression at the materno – foetal interface which might challenge the classical Th1/ Th2 dichotomy. J Reprod Immunol.

[17] Costeas PA, Koumouli A, Giantsiou-Kyriakou A, Papaloizou A, Koumas L (2004). Th2/Th3 cytokine genotypes are associated with pregnancy loss. Hum Immunol.

[18] D'Alfonso S, Rampi M, Rolando V, Giordano M, Momigliano-Richiardi P (2000). New polymorphisms in the IL-10 promoter region. Genes Immun.

[19] Crilly A, Hamilton J, Clark CJ, Jardine A, Madhok R (2003). Analysis of the 5′ flanking region of the interleukin 10 gene in patients with systemic sclerosis. Rheumatology (Oxford).

[20] Makhseed M, Raghupathy R, Azizieh F, Omu A, Al-Shamali E, Ashkanani L (2001). Th1 and Th2 cytokine profiles in recurrent aborters with successful pregnancy and with subsequent abortions. Hum Reprod.

[21] Austgulen R, Lien E, Liabakk NB, Jacobsen G, Arntzen KJ (1994). Increased levels of cytokines and cytokine activity modifiers in normal pregnancy. Eur J Obstet Gynecol Reprod Biol.

[22] Von Wollf M, Thaler CJ, Strowitzki T, Broome J, Stolz W, Tabibzadeh S (2000). Regulated expression of cytokines in human endometrium throughout the menstrual cycle: dysregulation in habitual abortion. Mol Hum Reprod.

[23] Hayakawa T, Takamura T, Hisada A, Abe T, Nomura G, Kobayashi K (2002). IL-6 gene polymorphism -174G/C does not contribute substantially to hyperlipidaemia and Type II diabetes mellitus in Japanese men. Diabetologia.

[24] Zhai R, Liu G, Yang C (2001). The G to C polymorphism at -174 of the interleukin-6 gene is rare in a southern Chinese population. Pharmacogenetics.

[25] Prigoshin N, Tambutti M, Larriba J, Gogorza S, Testa R (2004). Cytokine gene polymorphisms in recurrent pregnancy loss of unknown cause. Am J Reprod Immunol.

[26] Choi YK, Kwak-Kim J Cytokine gene polymorphisms in recurrent spontaneous abortions: a comprehensive review. Am J Reprod Immunol2008.

